# DEPTOR suppresses the progression of esophageal squamous cell carcinoma and predicts poor prognosis

**DOI:** 10.18632/oncotarget.7420

**Published:** 2016-02-16

**Authors:** Yan-Mei Ji, Xue-Feng Zhou, Jun Zhang, Xiang Zheng, Sheng-Bao Li, Zhi-Qiang Wei, Tao Liu, Dong-Liang Cheng, Ping Liu, Kuncheng Song, Tao Tan, Hua Zhu, Jia-Long Guo

**Affiliations:** ^1^ Department of Intensive Care Unit, Taihe Hospital, Hubei University of Medicine, Shiyan, People's Republic of China; ^2^ Department of Thoracic and Cardiovascular Surgery, Zhongnan Hospital of Wuhan University, Wuhan, People's Republic of China; ^3^ Department of Cardiothoracic Surgery, Taihe Hospital, Hubei University of Medicine, Shiyan, People's Republic of China; ^4^ Department of Gastroenterology, Taihe Hospital, Hubei University of Medicine, Shiyan, People's Republic of China; ^5^ Institute of Biomedical Research, Taihe Hospital, Hubei University of Medicine, Shiyan, People's Republic of China; ^6^ Department of Pathology, Taihe Hospital, Hubei University of Medicine, Shiyan, People's Republic of China; ^7^ Department of Surgery, Davis Heart and Lung Research Institute, The Ohio State University Wexner Medical Center, Columbus, Ohio, The United States

**Keywords:** DEPTOR, mTOR, esophageal squamous cell carcinoma, proliferation

## Abstract

As a naturally occurring inhibitor of mTOR, accumulated evidence has suggested that DEPTOR plays a pivotal role in suppressing the progression of human malignances. However, the function of DEPTOR in the development of esophageal squamous cell carcinoma (ESCC) is still unclear. Here we report that the expression of DEPTOR is significantly reduced in tumor tissues derived from human patients with ESCC, and the downregulation of DEPTOR predicts a poor prognosis of ESCC patients. In addition, we found that the expression of DEPTOR negatively regulates the tumorigenic activities of ESCC cell lines (KYSE150, KYSE510 and KYSE190). Furthermore, ectopic DEPTOR expression caused a significant suppression of the cellular proliferation, migration and invasion of KYSE150 cells, which has the lowest expression level of DEPTOR in the three cell lines. Meanwhile, CRISPR/Cas9 mediated knockout of DEPTOR in KYSE-510 cells significantly promoted cellular proliferation, migration and invasion. In addition, *in vivo* assays further revealed that tumor growth was significantly inhibited in xenografts with ectopic DEPTOR expression as compared to untreated KYSE150 cells, and was markedly enhanced in DEPTOR knockout KYSE-510 cells. Biochemical studies revealed that overexpression of DEPTOR led to the suppression of AKT/mTOR pathway as evidenced by reduced phosphorylation of AKT, mTOR and downstream SGK1, indicating DEPTOR might control the progression of ESCC through AKT/mTOR signaling pathway. Thus, these findings, for the first time, demonstrated that DEPTOR inhibits the tumorigenesis of ESCC cells and might serve as a potential therapeutic target or prognostic marker for human patients with ESCC.

## INTRODUCTION

Mammalian target of rapamycin (mTOR) is a downstream protein kinase of the PI3K/Akt signaling pathway. Mounting evidence has identified that abnormal activation of mTOR occurs in many types of cancer, which results an increase in protein synthesis responsible for growth, nutrient and energy signals that are essential for tumor progression [1, 2]. Thus, mTOR could be an attractive target for anticancer therapy, indeed, many mTOR inhibitors have been developed and are undergoing clinical trials to fight against various types of cancers, like sirolimus (CCI-779), everolimus (RADD001) and ridaforolimus (AP23573) [3–5].

DEPTOR is a recently identified mTOR binding protein that inhibits the kinase activities of both mTORC1 and mTORC2, two subcomplexes of mTOR [6]. Furthermore, the antitumor activity of DEPTOR has been confirmed in colorectal cancer [7], liver cancer [8], multiple myeloma [9] and pancreatic cancer [10]. Decreased expression of DEPTOR resulted the activation of AKT/mTOR pathway, which directly phosphorylated the downstream SGK1 and its substrate NDRG1, and thus promoted proliferation of tumor cells [11, 12]. In clinical investigations, downregualtion of DEPTOR has been found in pancreatic and colorectal cancer, which makes it a potential marker for prognosis of tumor patients [7, 10]. However, in other subsets of tumor like myeloma [6, 13, 14], thyroid carcinoma [15] and breast cancer [16], DEPTOR acts as an oncogene and positively correlates with poor survival of tumor patients, since DEPTOR overexpression simultaneously suppresses S6K1, a downstream molecule of mTOR, and thus relieves the feedback inhibition from mTOR to PI3K, boosting AKT activity for cancer cells survival [14, 15, 17]. The divergent impacts of DEPTOR in different cancers makes it vital to understand the detailed role of DEPTOR in specific cancer.

Esophageal squamous cell carcinoma (ESCC) ranks as the fifth most frequent cancer and the fourth common cause of cancer-related death in China (http://globocan.iarc.fr/Default.aspx). The World Health Organization (WHO) estimated that there were 197,472 cancer-associated deaths globally in 2012, with approximately 50% of total ESCC cases occur in China [18]. Most ESCC patients in China were diagnosed with advanced stages of the disease and the 5 year survival rate is only 4.4% [18]. Abnormal activation of mTOR occurs in ESCC patients that from different human population and range from 25% to 70% [19, 20]. mTOR inhibitors, like rapamycin and everolimus, inhibited proliferation, invasion, and induced apoptosis of ESCC cells *in vitro* [19, 21]. In addition, rapamycin alone or combined with cisplatin suppressed the tumor growth in tumor-bearing nude mice model [22].

Although a potential role for DEPTOR as an oncogene or a tumor suppressor has been investigated in different types of tumors [6–8, 10, 13], it has not been previously tested whether DEPTOR plays a role in the development of ESCC. Here we show that DEPTOR expression is significantly reduced in tumor tissues, and predicts a poor survival of ESCC patients. Retrospective study analysis further showed that DEPTOR negatively correlates with TNM stage and lymph node metastasis of ESCC patients. In *in vitro* studies, ectopic expression of DEPTOR in KYSE-150 ESCC cells that has a relative lower level of DEPTOR significantly suppressed cellular proliferation, migration and invasion, as well as inhibited the *in vivo* tumor growth in a nude mice model. Meanwhile, the tumor suppressive role of DEPTOR was also confirmed in another cell line KYSE-510 by knockout DEPTOR expression with CRISPER/Cas9 system, as KYSE-510 cells express a higher level of DEPTOR. Molecular analysis further revealed that DEPTOR inhibited the activation of AKT/mTOR pathway, indicating DEPTOR might control the progression of ESCC by downregulating this signaling pathway. Thus, our study suggests that DEPTOR inhibits the tumorigenesis of ESCC cells and might serve as a potential therapeutic target or prognostic marker for human patients with esophageal squamous cell carcinoma.

## RESULTS

### DEPTOR expression decreases tumor tissues derived from patients with ESCC

**Figure 1:**
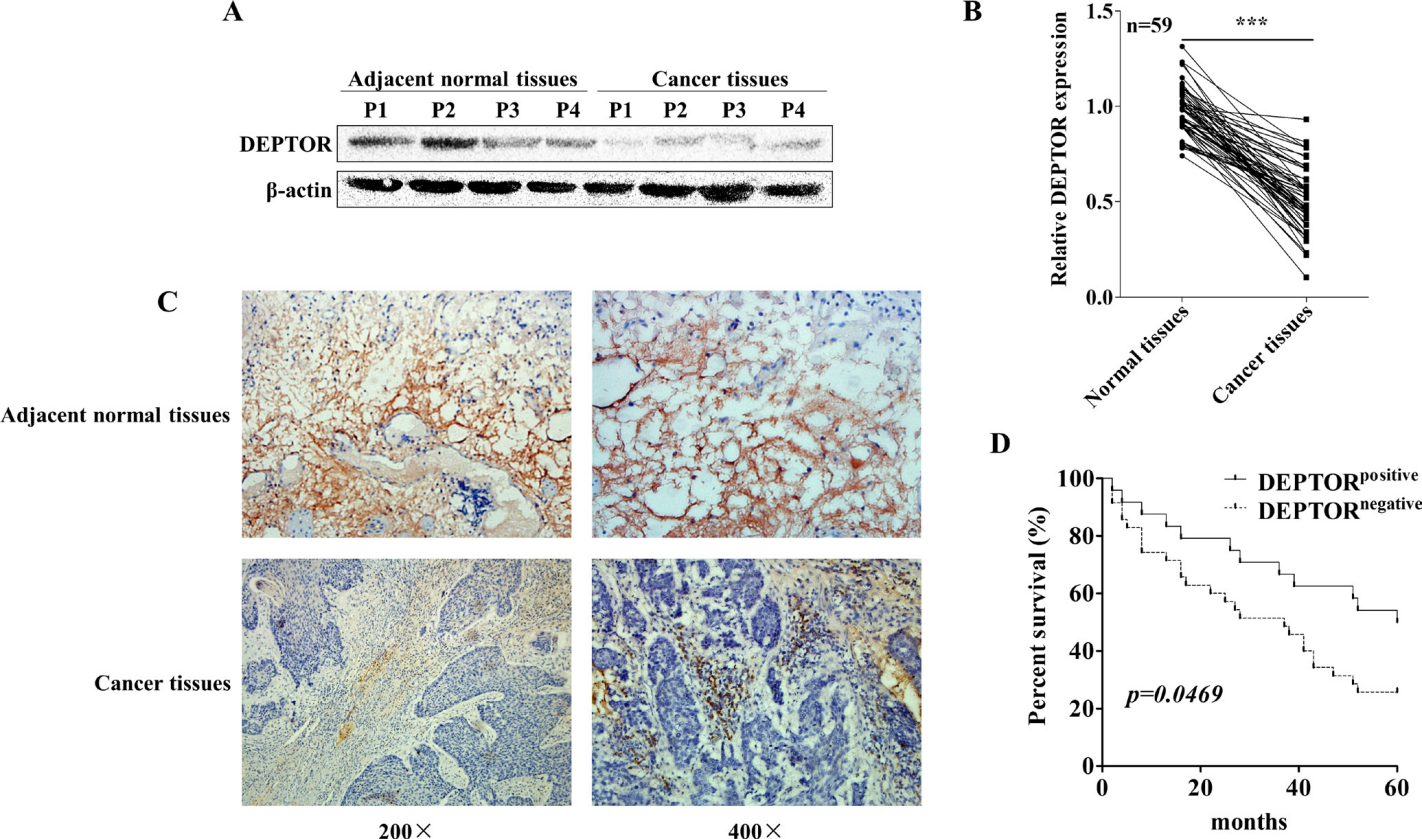
DEPTOR expression is decreased in human ESCC tissues and predicts a poor prognosis of ESCC patients (**A**) DEPTOR expression in ESCC tissues and non-cancerous adjacent tissues derived from human patients was detected by Western blotting, β-actin was used as the internal control. P1 means patient number 1. Western blotting results were quantified by ImageJ software and summarized in *right panel*. **p < 0.01. (**B**) mRNA expression of DEPTOR in samples from 59 ESCC patients was determined by qRT-PCR, GAPDH was used as an internal control and the date was analyzed by the 2^−ΔΔCt^ method. ***p < 0.0001. (**C**) Representative pathological images showed expression of DEPTOR reduced in ESCC samples. (**D**) DEPTOR staining in all samples were photographed and scored and grouped into two groups by qualified pathologists in a blind manner. The Kaplan-Meier survival curve showed that DEPTOR positive group displayed a better prognosis that DEPTOR negative group.

To determine the potential role of DEPTOR in progression of human ESCC, we firstly examined the expression of DEPTOR in cancer tissues from ESCC patients and paired adjacent non-cancerous tissues from the same patients. Western blotting analysis showed that protein expression of DEPTOR was significantly decreased in ESCC patients (Figure 1A). To further confirm our observations in a larger cohort, qRT-PCR was conducted to evaluate the mRNA level of DEPTOR in 59 ESCC patients admitted in Taihe Hospital Affiliated to Hubei University of Medicine, China. The result showed that mRNA expression of DEPTOR was significantly reduced in tumor tissues as compared to that in paired adjacent normal tissues in ESCC patients (Figure 1B, *p < 0.0001*). Thus, we confirmed that both DEPTOR protein and mRNA expression were decreased in ESCC tissues as compared with paired non-cancerous tissues.

### Downregualtion of DEPTOR in tumor tissue correlates with poor prognosis of ESCC patients

In order to further establish the correlation of DEPTOR expression with clinical prognosis, pathological slides from 59 ESCC patients were stained with DEPTOR antibody. Consistent with immunoblotting and qRT-PCR results, we found that DEPTOR is expressed in cytoplasm of non-cancerous cells, however its expression in paired tumor cells is greatly reduced (Figure 1C). As summarized in Table 1, expression of DEPTOR in ESCC was negatively correlated with TNM stage (*p = 0.0003*) and lymph node metastasis (*p = 0.010*), while not related to other clinicopathological characteristics, such as age (*p = 0.746*), gender (*p = 0.854*) and histologic grade (*p = 1.000*, namely differentiation). Furthermore, Kaplan-Meier survival analysis demonstrated that patients with high DEPTOR expression levels had a higher five year survival rate than that with lower DEPTOR expression in a retrospective cohort study (Figure 1D, *p = 0.0469*).

**Table 1:** Clinicopathological characteristics and DEPTOR expression in patients with ESCC

Characteristic	DEPTOR			
	Negative	Positive	*X*^2^	*p* value
Age			0.105	*0.746*
≤ 53	19	12		
≥ 53	16	12		
Gender			0.034	*0.854*
Male	27	19		
Female	8	5		
Tumor size			1.787	*0.409*
≤ 3 cm	7	6		
3–5 cm	15	13		
> 5 cm	13	5		
Tumor location			0.185#	*1.000*
Upper	5	3		
Middle	14	9		
lower	16	12		
TNM stage			8.866	0.003*
I–II	11	17		
III–IV	24	7		
Lymph node metastasis			6.602	0.010*
Yes	19	5		
No	16	19		
Differentiation^a^			0.125#	*1.000*
Well	6	4		
Moderate	21	15		
poor	8	5		

ESCC cases (*n* = 59) were categorized as DEPTOR-positive (*n* = 24) and DEPTOR-negative (*n* = 35). χ test was used to test the difference expression of DEPTOR.

# If cells have expected count less than 5, Fisher's Exact test was used.

* *p* < 0.05.

a Well, well differenciated squamous cell carcinoma; Moderate, moderately differenciated squamous cell carcinoma; poor, poorly differenciated squamous cell carcinoma.

### DEPTOR regulates proliferation, migration and invasion of ESCC cells

**Figure 2:**
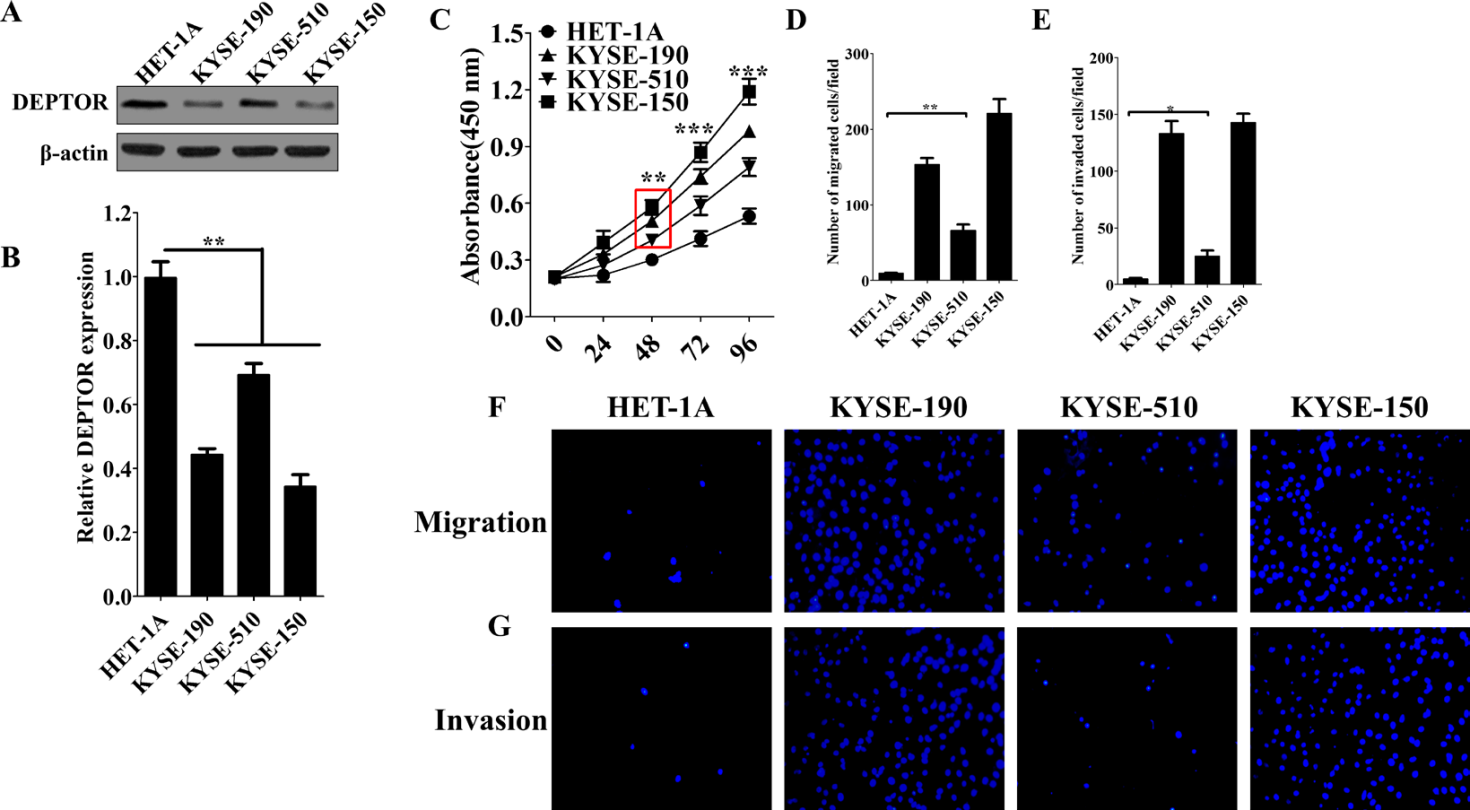
Expression of DEPTOR is differentially reduced in ESCC cells and may be involve in regulating tumor progression (**A**) Protein and (**B**) mRNA expression of DEPTOR was reduced in three ESCC cell lines, KYSE-150, KYSE-510, KYSE-190, as compared to normal control cells, HET-1A. Cell proliferation rate (**C**), Cell migration (**D**, **F**) and invasion (**E**, **G**) abilities in KYSE-150 cells are highest among three ESCC cell lines. Six random fields were photographed (F, G) and summarized (D, E) in each group. Data presented as Mean ± SD, *n* ≥ 3 independent experiments. *p < 0.05, **p < 0.01, ***p < 0.0001.

In order to study a potential role of DEPTOR in regulation of tumor progression, several ESCC cell lines, KYSE-150, KYSE-510 and KYSE-190, and normal esophageal squamous epithelial cell HET-1A were used. Consistent to our observations in patients, the protein and mRNA levels of DEPTOR were reduced in ESCC cell lines as compared to HET-1A cells (Figure 2A and 2B). Among ESCC cell lines, DEPTOR expression was lowest in KYSE-150 cells and highest in KYSE-510 cells (Figure 2A). We then tested whether the differential expression of DEPTOR will lead to differences in proliferation, migration and invasion among these cell lines. Cellular proliferation analysis showed that KYSE-150 cells proliferated significantly faster than KYSE-190 and KYSE-510 cells (Figure 2C, *p < 0.0001*), and a same trend was also observed in evaluation of cell migration (Figure 2D and 2F) and invasion (Figure 2E and 2G). Thus, we hypothesized that DEPTOR might be involved in regulation of proliferation, migration and invasion of ESCC cells.

**Figure 3:**
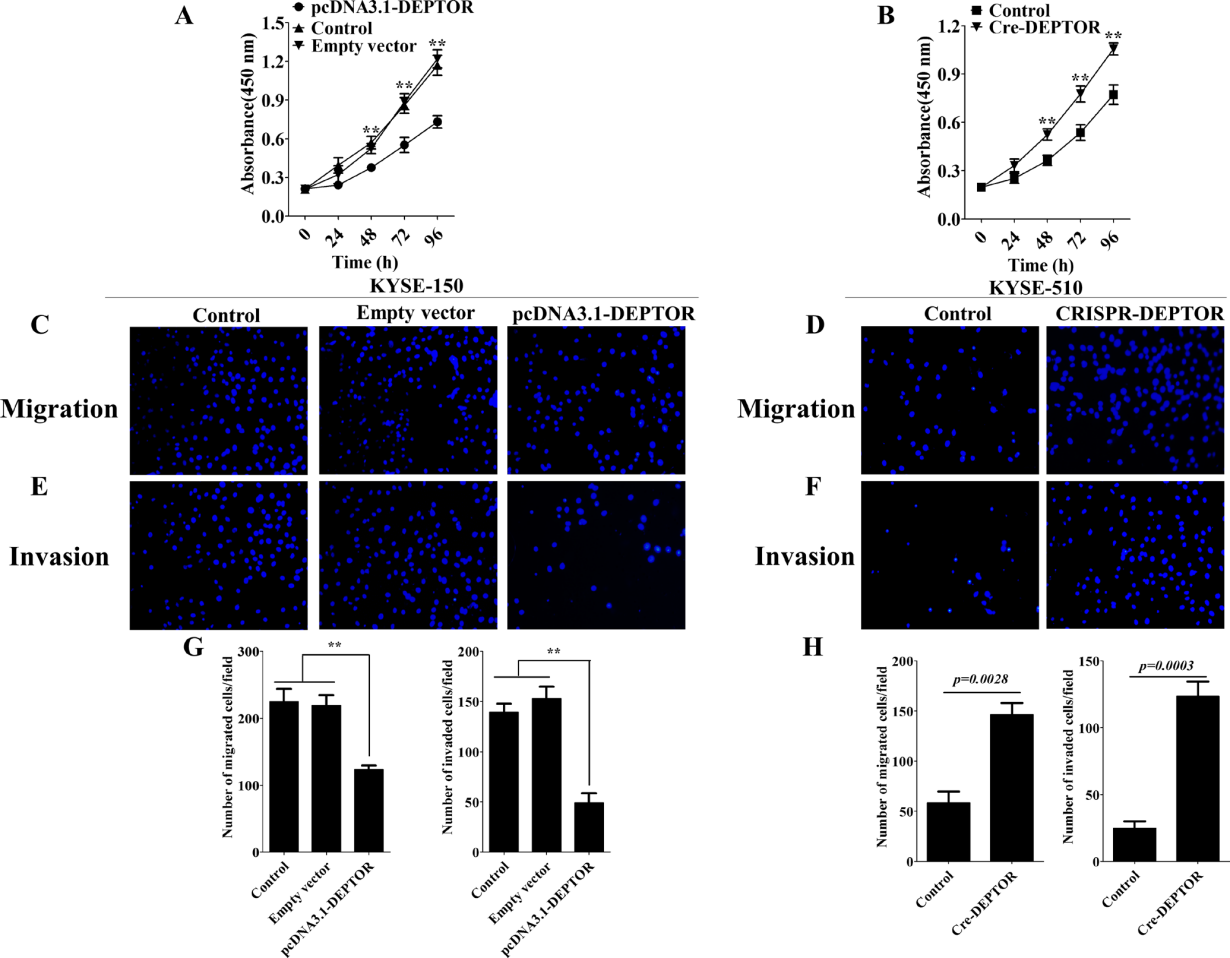
DEPTOR inhibits proliferation, migration and invasion of ESCC cells KYSE-150 cells were transfected with plasmid to generate stable DEPTOR overexpression cell line (pcDNA3.1-DEPTOR), while KYSE-510 cells were treated with CRISR/Cas9 system to generate DEPTOR knockout cell line (CRISPR-DEPTOR). Overexpression of DEPTOR led to reduced proliferation rate (**A**), migration (**C** and **G** *left panel*) and invasive (**E** and G *right panel*) abilities, while knockout of DEPTOR caused enhanced proliferation rate (**B**), migration (**D** and **H** *left panel*) and invasive (**F** and H *right panel*) abilities in ESCC cells. Data presented as Mean ± SD, *n* = 3 independent experiments. **p < 0.01.

In order to further test our hypothesis, we generated stable cell lines that either overexpressing DEPTOR or genetic ablation of endogenous expression of DEPTOR. Since KYSE-150 expresses lowest endogenous level of DEPTOR among the three cell lines, we stably overexpressed DEPTOR in KYSE-150 cells (pcDNA3.1-DEPTOR). For the same consideration, we treated KYSE-510 cells, which expresses highest level of DEPTOR, with CRISPR/Cas9 system to knockout of DEPTOR (CRISPR-DEPTOR). After generation of cell lines, we then performed proliferation, migration and invasion experiments by using genetically modified cells as compared with parental cells. As shown in Figure 3A and 3B, pcDNA3.1-DEPTOR displayed a reduced cell proliferation rate as compared to that of KYSE-150 parental cells and empty vector transfected cells, while CRISPR-DEPTOR cells proliferated significantly faster than control KYSE-510 cells. Furthermore, pcDNA3.1-DEPTOR cells also showed reduced migration (Figure 3C and 3G *left panel*) and invasive (Figure 3E and 3G *right panel*) capacities, while CRISPR-DEPTOR cells are more aggressive than the control KYSE-510 cells in terms of migration (Figure 3D and 3H *left panel*) and invasion (Figure 3F and 3H *right panel*). Thus, these results suggested DEPTOR indeed regulates cell proliferation, migration and invasion in ESCC cells.

### DEPTOR suppresses the activation of AKT/mTOR pathway in ESCC cells

**Figure 4:**
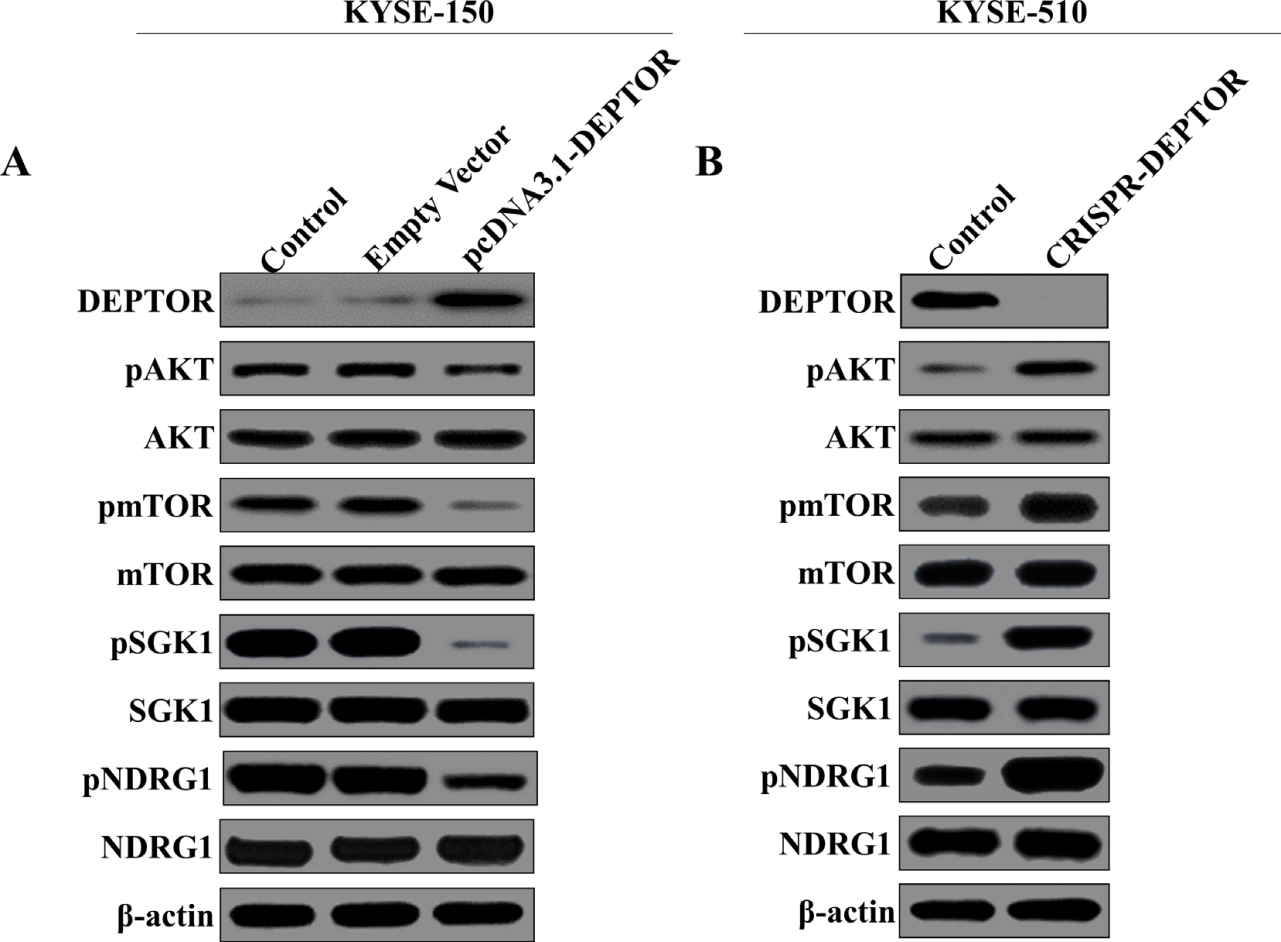
DEPTOR inhibits AKT/mTOR signaling pathway in ESCC cells Overexpression of DEPTOR led to inhibition of AKT/mTOR signaling pathway as evidenced by downregulation of phosphorylation of key components in this pathway (**A**), such as AKT, mTOR, SGK1, NDRG1, while knockout of DEPTOR led to activation of the same pathway (**B**).

In order to determine the molecular mechanisms underlying DEPTOR mediated tumor suppression, AKT/mTOR signaling pathway was tested by western blotting in our established cells lines. As shown in Figure 4A, DEPTOR expression was significantly increased in KYSE-150 cells as compared with untreated cells or empty vector-transfected cells. mTOR has been reported to promote cell survival via directly phosphorylating SGK1 and its downstream NDRG1 [11, 12]. As a natural inhibitor of mTOR, ectopic expression of DEPTOR resulted in the deactivation of AKT/mTOR pathway, as it inhibited the phosphorylation of AKT, mTOR, SGK1 and NDRG1 (Figure 4A). And in KYSE-510 cells, knockout of DEPTOR expression significantly enhanced the activation of AKT/mTOR pathway (Figure 4B). Thus, we demontrated that DEPTOR negatively regulated the activation of AKT/mTOR pathway which may be responsible for its tumor suppressive function.

### DEPTOR inhibits the growth of ESCC cells *in vivo*

**Figure 5:**
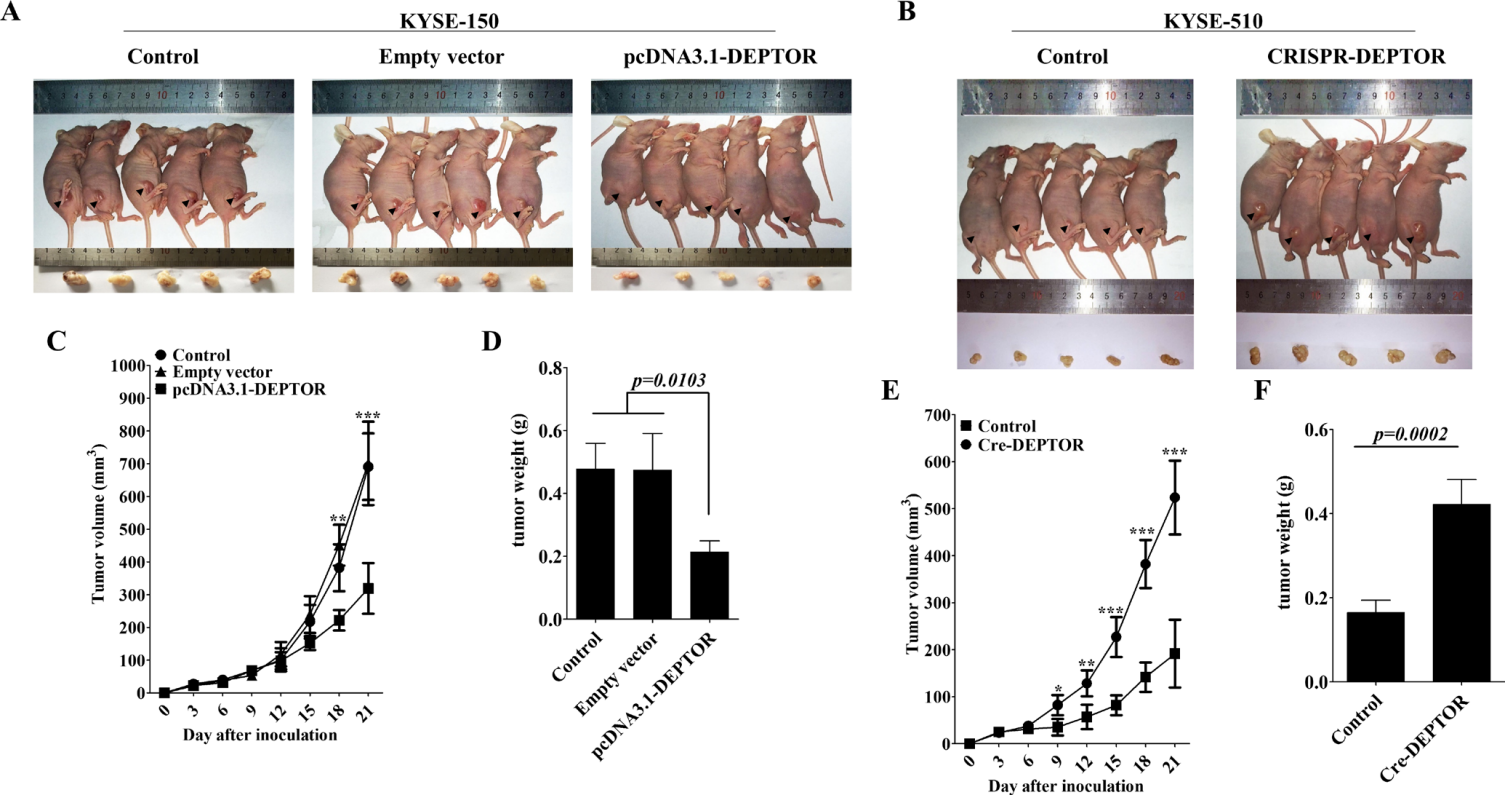
DEPTOR inhibits *in vivo* tumor growth of ESCC cells Six-to-eight week's old female BALBc/nude mice were subcutaneously injected with KYSE-150 cells or KYSE-510 cells in the flank of mice (1 × 10^6^) in a volume of 100 μl at the time of inoculation. The mice were sacrificed (**A, B**) and the tumors were dissected and weighted (**D, F**) on the 21th day after inoculation. Tumor size was measured by using a digital caliper every three days after injection (**C, E**). Data presented as Mean ± SD, five mice for each group. *p < 0.05, **p < 0.01, ***p < 0.0001.

To further test the role of DEPTOR in regulation of ESCC cell growth *in vivo*, nude mice were inoculated with untreated KYSE-150 cells or pcDNA3.1-DEPTOR cells. The volume of tumor xenografts were monitored every 3 days and at day 21 after injection, they were removed for photograph and weight measurement. The results showed that overexpression of DEPTOR significantly suppressed growth of xenografts in nude mice from 15 days after inoculation (Figure 5A and 5C) as well as the tumor weights for about 55.2% at the end of study (Figure 5D, *p = 0.0103*). Similarly, in KYSE-510 cells, ablation of DEPTOR significantly promoted tumor growth in nude mice as compared with normal KYSE-510 cells (Figure 5B, 5E and 5F). Therefore, these results suggested that DEPTOR can regulate ESCC tumor growth *in vivo*.

## DISCUSSION

As an mTOR binding protein that normally functions to inhibit the mTORC1 and mTORC2 pathways, DEPTOR is considered as a tumor suppressor protein with the reason that mTOR activity is mostly hyperactivated in many human tumors [4, 23, 24], Indeed, down-regulation of DEPTOR has been found in many types of human cancers [7–10, 17]. However, the function of DEPTOR in tumorigenesis is still controversial, as DEPTOR has also been found to be overexpressed in many other tumor types including breast cancer, chronic myeloid leukemia and multiple myeloma [6, 13, 16]. Therefore, the potential role of DEPTOR as an oncogene or a tumor suppressor may be cell context or tissue specific. In this study, we firstly reported that DEPTOR expression is significantly decreased in tumor tissues of ESCC patients, and predicts a poor five-year survival rate. Detailed analysis further revealed that DEPTOR is significantly correlated with TNM stage and Lymph node metastasis. However, as we only collected 59 ESCC samples in our study, we did not observe significant correlation between DEPTOR expression and tumor differentiation, this may be own to the restrict in number of some subpopulations (like well differentiation group only include 4 samples), and the correlation analysis may be need to be reexamined in a large patient population.

In some cases of ESCC patients that recruited into our study, DEPTOR expression just showed a minor difference between adjacent normal tissue and cancer tissue, this may be own to that the regulation of DEPTOR is complicated in different individual. Just like in multiple myeloma, one study reported that mTORC1 and mTORC2 inhibitors inhibit tumor growth, including DEPTOR [9], whereas another study revealed that DEPTOR silence induces cytoreductive effects on multiple myeloma (MM) cells [13]. This may be due to that about 21–28% of human MMs were shown to harbor Cyclin D1/D3 or c-MAF/MAFB translocations, and possess copy number gains and associated expression increases of the genes within a 6 Mb region of chromosome 8q24 that contains DEPTOR. In these cells, high DEPTOR expression is necessary to maintain PI3K and Akt activation, as DEPTOR overexpression suppresses the expression of S6K1, a downstream molecule of mTOR, and thus relieve feedback inhibition from mTORC1 to PI3K signaling, a reduction in DEPTOR levels leads to apoptosis of these cells [6, 14].

Aberrant activation of the mTOR pathway has been identified in ESCC, and mTOR specific inhibitors, like everolimus, rapamycin and PP242, exert therapeutic effects both as a single agent and in combination with cisplatin [19, 21, 22, 25]. Among these inhibitor, PP242 is a dual mTORC1 and mTORC2 inhibitor that similar with DEPTOR, which has been reported to suppress proliferation, metastasis, and angiogenesis of gastric cancer cells through inhibition of the PI3K/AKT/mTOR pathway [26, 27]. Furthermore, it also effectively suppressed proliferation and induced apoptosis in ESCC cells [25]. As far as the role of DEPTOR in ESCC progression, we found that ectopic expression of DEPTOR inhibited the activation of AKT/mTOR pathway, and suppressed proliferation, migration, and invasion as well as *in vivo* tumor growth of ESCC cells with relatively low DEPTOR expression. And the inhibitory role of DEPTOR was also identified with DEPTOR knockout. However, whether DEPTOR directly depends on repressing AKT/mTOR pathway to inhibit ESCC progression remains need to be identified in future.

In breast cancer, studies showed that DEPTOR expression is repressed by EMT programs in estrogen receptor α negative cells, and also decreased in cancer stem cells or tumor tissues of patients with aggressive breast cancers, however it is essential for the growth and Chemo-resistance of tumor cells [16, 28]. Indeed, one study conducted by Hua et al. [10] found that DEPTOR suppresses anchorage-dependent or independent growth of pancreatic cancer cells. Anchorage-independent is a classic measurement of tumor cell with stem cell-like phenotype [29, 30]. In our study, we have noticed that DEPTOR inhibits the proliferation, migration and invasion of ESCC cells, future studies need to delineate whether DEPTOR regulate chemo-resistance in ESCC cells, as well as the role of DEPTOR in cancer stem cells of ESCC. In addition, there are some limitations in our study, for example, CRISPR/Cas9 system has been reported to have “off-targets”. Although through our initial screening, we haven't found predicted off-targets in our cell lines, we cannot rule out there are other off-targets which were not predicted by the prediction software. Therefore, our future studies will try to use multiple guide RNA sequences and to compare whether the effects of knockout of DEPTOR is consistent among individual probe. Collectively, our findings implicate that DEPTOR is an essential inhibitor during ESCC development and may serve as a potential biomarker to predict prognosis of the human patients with ESCC.

## MATERIALS AND METHODS

### Patients and tissue samples

All research involving human participants was approved by the Ethics Committees of Taihe Hospital Affiliated to Hubei University of Medicine, China. Written informed consents have been obtained from all participants. Fifty-nine pathologically diagnosed biopsy specimens and paired adjacent normal tissues were acquired from patients with ESCC. The patients' clinicopathological characteristics are detailed in Table 1. Biopsy samples of these 59 patients were cut into 5 μm slices for DEPTOR immunohistochemical staining and then used for the retrospective cohort analysis of survival data of five year follow-up.

### Cell cultures

Normal esophageal squamous epithelial cell line HET-1A was obtained from American Type Culture Collection (ATCC, Manassas, USA), and cultured in BEGM that recommended by ATCC at 37°C in a humidified atmosphere with 5% CO_2_. Human ESCC cell lines (KYSE150, KYSE190, KYSE510) were cultured in RPMI 1640 and Ham F12 mixed (1:1) medium containing 5% fetal bovine serum (Gibco) at 37°C in a 5% CO2 air atmosphere as mentioned before [31].

### Immunohistochemical staining

Biopsy samples were fixed in 4% formalin, and embedded in paraffin. Tissue slices that were 5-μm thick were cut for H & E staining and examined under a microscope. Immunohistochemistry was performed using the Vectastain ABC Kit (Rabbit IgG, Vector Laboratories). The rabbit polyclonal DEPTOR antibody (Abcam) was used at 2 μg/ml (1:500) as primary antibody. Slices were developed with DAB and counterstained with hematoxylin. For immunohistochemistry assessment, the entire tissue section was scanned and scored by two independent pathologist. The scores were calculated as Lai et al. described [7] and higher than 1 was considered positive.

### Western blotting

Protein of tissue samples or cell lines was extracted by using Total Protein Extraction Kit (Millipore, Darmstadt, Germany). The protein concentration was determined using Bradford Protein Assay kit (Beyotime, shanghai, China). Equivalent proteins were denatured in protein loading buffer, loaded onto 10% SDS-PAGE gels, and subsequently transferred to polyvinylidene difluoride (PVDF) membranes (Millipore, Billerica, MA) by electroblotting. The PVDF membranes were blocked with 5% nonfat milk in TBST buffer for 1 h and incubated overnight at 4°C with antibody against DEPTOR (Abcam, 1:1000), Akt (Cell Signaling, 1:2000), pAkt (Ser473) (Cell Signaling, 1:1000) mTOR (Cell Signaling, 1:1000), pmTOR (Ser2448) (Cell Signaling, 1:1000), SGK1 (Cell Signaling, 1:1000), pSGK1(Ser422) (Abcam, 1:1000), NDRG1 (Cell Signaling, 1:1000), pNDRG1 ((Thr346) (Cell Signaling, 1:1000) and β-actin (Cell Signaling, 1:5000). Signals were detected using ECL detection reagent (Millipore) following the manufacturer's instructions.

### Quantitative real-time PCR (qRT-PCR)

RNA was extracted from tissue samples using TRIzol (Invitrogen) following the manufacturer's protocol. For DEPTOR mRNA detection, the forward primer is 5′-TTTGTGGTGCGAGGAAGTAA-3′, and the reverse primer is 5′-CATTGCTTTGTGTC ATTCTGG-3′. GAPDH was used as an internal control: Forward: 5′-CTC TCTGCTCCTCCTGTTCGAC-3′, Reverse: 5′-TGAGCG ATGTGGCTCGGCT-3′ [6]. The ABI StepOne Plus (Applied Biosystems, Foster City, CA) was used to perform the amplification reaction. Each experiment was performed in triplicate. And the date was analyzed by the 2^−ΔΔCt^ method.

### Establishment of stably transfected cells

The cDNA for DEPTOR (NCBI gene symbol DEP.6; DEPDC6) was amplified by PCR from HET-1A cells, and the product was subcloned into the BamH I and Hind III sites of pcDNA3.1 (Invitrogen, CA, USA) to generated DEPTOR overexpression plasmid [6, 32]. KYSE-150 cells was transfected with pcDNA3.1-DEPTOR recombinant plasmid or empty vector using Lipofectamine 2000 according to the manufacturer's instructions (Invitrogen, USA) and retained in medium containing 10% FBS and 1 μg/ml puromycin to select the stable transfected cells.

### CRISPR/Cas9 mediated DEPTOR knockout

2 × 10^5^ KYSE-510 cells in 3 ml antibiotic-free medium were plated in 6-well plate. 24 h later, after cells reach 80% confluence, CRISPR/Cas9 KO Plasmid and DEPTOR HDR Plasmid (Santa Cruz Biotechnology, Inc.) were mixed at equivalent ratio and then co-transfected into KYSE-510 cells using Lipofectamine 2000 according to the manufacturer's instructions. 48 h post-transfection, aspirate the medium and replace with fresh medium containing puromycin (1 μg/ml) to select and establish the stable transfected cells.

### Cell proliferation assay

Cell proliferation assays were carried out with a Cell Counting Kit-8 (Beyotime, shanghai, China) [33]. Cells were plated in 96-well plates at approximately 1 × 10^4^ cells per well. The numbers of cells per well were detected by the absorbance (450 nm) of reduced WST-8 at the indicated time points. The absorbance (450 nm) was measured by using SpectraMax^®^ i3x microplate reader (Molecular Devices, Sunnyvale, CA).

### Cell migration assay

Cell migration was examined in Boyden chamber system as described previously [34]. Cells that suspended in serum-free medium were added to the upper chamber (8-μm pore size, Corning Costar), and the chamber was placed in 24-well dishes. 10% FBS-medium were added in the lower chamber. Migration was permitted for 24 h, and cells were then fixed with 4% formaldehyde and stained with 4′,6-dia-midino-2-phenylindole (DAPI). The numbers of invasive cells were obtained by counting five fields and represented the average of three independent experiments.

### Cell invasion assay

Transwell inserts that precoated with Matrigel (8 μm pore; Corning Costar) were placed into the 24-well plate, a total of 1 × 10^5^ cells in 0.5% FBS-medium were seeded in the upper chamber and 10% FBS-medium were added in the lower chamber. After 24 h incubation, cells on the top surface of the insert were removed by wiping with a cotton swab. The cells that had invaded the bottom side of the membrane were fixed with 4% formaldehyde and stained with DAPI. The numbers of invasive cells were obtained by counting five fields per membrane and represented the average of three independent experiments.

### Xenograft mouse model

Six-to-eight week's old female BALBc/nude mice were purchased from Laboratory Animal Center of Wuhan University (Wuhan, China) and maintained in the cages under sterile conditions with a specific pathogen-free environment. KYSE-150 cells or KYSE-510 cells were subcutaneously injected in the flank of mice (1 × 10^6^) in a volume of 100 μl at the time of inoculation [35]. Tumor size was measured by external caliper measurement every three days after injection, and tumor volume was calculated as 1/2 × (tumor length) × (tumor width)^2^ [36]. The mice were sacrificed and the tumors were dissected and weighted on the 21th day after inoculation.

### Statistical analysis

All statistical analyses were performed using Graphpad Prism V.5.00 software (GraphPad Software, San Diego CA, USA). Statistical significance was determined at *p < 0.05*, and tests were two sided. Pearson's χ^2^ test or Fisher's exact test were used to test the difference between DEPTOR expression and clinicopathologic characteristics. Comparison between two groups for statistical significance were performed with unpaired Student's *t* test. For more groups, one-way ANOVA followed by Neuman-Keuls post hoc test was used. Survival probabilities were calculated using the Kaplan-Meier method. Differences in survival between patient subgroups were analyzed using log-rank (Mantel-Cox) Test.
